# Opposite diacylglycerol enantiomeric specificities of Arabidopsis DGAT1 and DGAT2 reveal distinct roles in TAG synthesis

**DOI:** 10.1093/plphys/kiag234

**Published:** 2026-04-21

**Authors:** Chinnu Ann Jaison, Joachim Björklund, Sten Stymne, Kamil Demski, Per Hofvander, Ida Lager

**Affiliations:** Department of Plant Breeding, Swedish University of Agricultural Sciences, Alnarp SE-230 53, Sweden; Centre for Analysis and Synthesis, Department of Chemistry, Lund University, Lund SE-221 00, Sweden; Department of Plant Breeding, Swedish University of Agricultural Sciences, Alnarp SE-230 53, Sweden; Department of Plant Breeding, Swedish University of Agricultural Sciences, Alnarp SE-230 53, Sweden; Department of Plant Breeding, Swedish University of Agricultural Sciences, Alnarp SE-230 53, Sweden; Department of Plant Breeding, Swedish University of Agricultural Sciences, Alnarp SE-230 53, Sweden

## Abstract

Triacylglycerol (TAG) serves as the primary storage lipid in plants, essential for seed germination and early seedling development. Acyl-CoA:diacylglycerol acyltransferase (DGAT) enzymes catalyze the last step in TAG synthesis by converting diacylglycerol (DAG) to TAG. Although functionally conserved across species, DGATs exhibit variations in substrate preference, among their other properties, highlighting their influence on fatty acid (FA) composition in TAG. In this study, we investigated the biochemical properties of Arabidopsis DGATs expressed in yeast with a primary emphasis on understanding the specificity and selectivity of these enzymes on different acyl acceptors and donors. One aim of the study was to investigate if the FA composition of TAG is due to DGAT selectivity in addition to being influenced by the distinct DAG pools (spatially separated de novo synthesized and phosphatidylcholine [PC]-derived DAG). Our findings showed that Arabidopsis (*Arabidopsis thaliana*) DGAT1 preferentially selects PC-derived DAGs. Further, we also report a new method for synthesizing *sn-2,3-*DAG that we used to study DGAT enantiomeric specificity. The results revealed that DGAT1 is specific toward the *sn-1,2-*DAG enantiomer whereas DGAT2 only utilizes *sn-2,3*-DAG, a substrate that is not directly involved in the Kennedy pathway. DGAT2 has so far not shown significant involvement in de novo TAG synthesis and thus our findings indicate a possible function for DGAT2 in Arabidopsis in TAG remodeling.

## Teaser

Arabidopsis diacylglycerol acyltransferases DGAT1 and DGAT2 show nonredundant and opposite enantiomeric specificity for diacylglycerol enantiomers.

## Introduction

Seed oils are a vital source of dietary fats and poses significant pharmacological value for human consumption. They are one of the major compounds in demand within oleochemical industries, serving as an essential constituent in paints, lubricants, biofuel, cosmetics, and polymers (Durrett et al. 2008; Parchuri et al. 2024). The primary component in seed oil is triacylglycerol (TAG), and its end-use is primarily influenced by the fatty acid (FA) composition of the TAG. More than 450 different FAs have been found in seed oils. Many of these are unusual FAs that have unique applications (Ohlrogge et al. 2018; Demski et al. 2019; Parchuri et al. 2024).

In plants, the de novo TAG biosynthesis occurs in the endoplasmic reticulum (ER). In this pathway, glycerol-3-phosphate (G3P) undergoes sequential acylation at the glycerol backbone by FAs, supplied by acyl-CoA. This process is catalyzed by glycerol-3-phosphate acyltransferase, lysophosphatidic acid acyltransferase, phosphatidic acid phosphohydrolase, and diacylglycerol (DAG) acyltransferase, each catalyzing a specific step in TAG synthesis (Liu et al. 2012; Bhandari and Bates 2021). The lipid biosynthesis process is, however, more complex and involves multiple other enzyme-catalyzed reactions, including reciprocal DAG to phosphatidylcholine (PC) flux regulated by phosphatidylcholine:diacylglycerol cholinephosphotransferase (PDCT). Moreover, FA desaturations occur when FAs are attached to PC by the fatty acid desaturases (FAD) enzyme introducing multiple double bonds in the acyl chain (Wang et al. 2012; Bates 2016). By this reaction, PC-derived DAG are produced in many plants with high amounts of polyunsaturated fatty acids (PUFA) such as in Arabidopsis (*Arabidopsis thaliana*), where it is the main DAG substrate for TAG synthesis (Bates and Browse 2011). A portion of these PUFAs are transferred from PC to the acyl-CoA pool by acyl exchange catalyzed by the acyl-CoA: lysophosphatidylcholine acyltransferase (LPCAT) enzyme (Bates 2016). In addition to DGATs, phosphatidylcholine:diacylglycerol acyltransferase (PDAT) catalyzed TAG synthesis can occur with DAG and PC as substrates (Dahlqvist et al. 2000).

DGATs are acyl-CoA–dependent enzymes that play a key regulatory role in determining the FA composition of TAG in the G3P pathway by catalyzing the final acylation step in the synthesis. DGATs have generally been believed to catalyze the acylation of FA obtained from acyl-CoA to the *sn-3* position of the glycerol backbone of DAG (Shockey et al. 2006; Li et al. 2010). Understanding the specificity and selectivity of DGAT for both its substrates, DAG (acyl acceptor) and acyl-CoA (acyl donor), gives valuable insight into the composition of TAG in seeds and how it is regulated in different plants (Liu et al. 2012). Two distinct, nonhomologous DGATs, both membrane-bound in the ER, have been identified and characterized in nearly all eukaryotes. Depending on the eukaryotic species, the 2 DGATs can have both redundant and non-redundant roles in TAG synthesis in different tissues (Yen et al. 2008; Turchetto-Zolet et al. 2011). DGAT1, a member of the membrane-bound O-acyltransferase (MBOAT) family, is well-characterized and recognized as the major TAG-synthesizing enzyme in many plants (Hobbs et al. 1999; Hatanaka et al. 2022). DGAT2 belongs to the evolutionarily unrelated monoacylglycerol acyltransferase (MGAT) family and is known for its involvement in TAG biosynthesis, including unusual fatty acids, such as hydroxy FA in Castor (*Ricinus communis*) (Burgal et al. 2008; Demski et al. 2022), *Physaria fendleri* (Parchuri et al. 2024) and epoxy FA in *Vernonia galamensis* (Li et al. 2010). Characterizing DGAT properties is vital for understanding TAG accumulation processes, enabling the development of plant varieties with tailored FA profiles and enhanced TAG yields. Such modifications are highly relevant to the chemical and biofuel industries, where there is a growing demand for sustainable alternatives to carbon fossil sources that are used to produce chemicals in products like cosmetics, lubricants, and paints. Hence, replacing synthetic chemicals with plant-derived alternatives offers a promising eco-friendly solution to displace use of fossil fuels (Demski et al. 2019; Regmi et al. 2020).

In this work, we investigated the acyl-CoA and DAG specificities and selectivity, as well as the enantiomeric preference of DAG species by Arabidopsis DGAT1 and DGAT2 enzymes. The DGAT genes were overexpressed in a yeast mutant strain devoid of TAG synthesis. Arabidopsis, belonging to the Brassicaceae family, is the most extensively studied plant model species. There are many published studies using either knockouts or over-expressors of DGATs, including gene expression analysis and lipidomics data of the seed TAG (Taylor et al. 1995; Regmi et al. 2020; Neumann et al. 2024). However, reports on in-depth biochemical characterization of Arabidopsis DGAT1 and DGAT2 enzymes are lacking. Major FAs observed in Arabidopsis TAG are 16:0, 18:0, 18:1, 18:2, 18:3, and 20:1 (Katavic et al. 1995). The de novo DAGs produced contain 16:0 and 18:1 FA derived from plastid, whereas PUFAs derived by acyl exchange of acyl-CoA with PC, catalyzed by LPCAT. PC is the site for oleate desaturation of 18:1 to 18:2 and 18:3 FA and is then converted to DAG to form PC-derived DAG by PDCT (Bates 2016). [^14^C]Glycerol labeling of Arabidopsis developing seeds revealed that PC-derived DAG (containing higher PUFAs levels than de novo formed DAG) is utilized for TAG synthesis by DGAT1 rather than the de novo synthesized DAG (Bates and Browse 2011). A third, slowly producing bulk DAG pool was also identified and is utilized by PDAT and DGAT2 (Regmi et al. 2020). These results suggest that DAG consists of several spatially separate pools. In addition to utilizing different DAG pools, the acyl acceptor selectivities of DGATs can also influence the fatty acid composition of the produced TAG. Knockout of the DGAT1 gene in Arabidopsis resulted in a significant reduction (over 20%) in oil content, with decreased levels of 18:1 FA and increased levels of PUFA compared with the Col-0 wild type (Zhang et al. 2009; Regmi et al. 2020). It has been reported that in the absence of either PDAT or DGAT2 in Arabidopsis, there is no significant reduction in the oil content (Mhaske et al. 2005; Zhang et al. 2009), making DGAT1 the primary contributor to TAG biosynthesis. However, silencing PDAT in *dgat1* knockouts further reduces the oil content by 80% (Zhang et al. 2009; Xu et al. 2012), a phenomenon not observed in *dgat1-dgat2* double knockout Arabidopsis plants, suggesting that PDAT is the second major contributor to TAG synthesis (Regmi et al. 2020).

The role of DGAT2 in plants, including its involvement in the metabolism of substrates with unusual FAs in some species, is an interesting area to investigate further. Recent progress in lipid metabolism studies has uncovered a previously unrecognized mechanism governing TAG composition in oilseeds, TAG remodeling. In this mechanism, newly produced TAG can be hydrolyzed by lipase enzymes. This hydrolysis removes FA from either the *sn-1* or *sn-3* position in TAG, leading to the formation of 2 enantiomeric DAG species (*sn-1,2-*DAG and *sn-2,3-*DAG) (Bates and Shockey 2025). In vivo metabolic tracing studies in developing seeds of *P. fendleri* identified the TAG remodeling pathway (Bhandari and Bates 2021). Later, Parchuri et al. (2024) showed that *Pfe*DGAT1 acylates *sn-1,2*-DAG and *Pfe*DGAT2 acylates *sn-2,3*-DAG and also identified a *Pfe*TAGL1 lipase that hydrolyzes the TAG at *sn-1* position to form *sn-2,3* enantiomers of DAG. This raises the question of whether DGATs of other plants can also utilize *sn-2,3-*DAG as a substrate to resynthesize TAG from this DAG pool. The only evidence to date is in *P. fendleri* DGATs and indicates differential preference, with DGAT1 favoring *sn-1,2-*DAG and DGAT2 favoring *sn-2,3-*DAG (Parchuri et al. 2024; Bates and Shockey 2025). These results unveil a previously uncharacterized mechanism in TAG biosynthesis, highlighting the need for further studies on DAG enantiomer preference of DGATs in other plant species and how that would affect the overall FA composition of TAG. To our knowledge there are no reports where pure *sn-2,3-*DAG enantiomer has been used in DGAT assays or other enzyme assays, probably due to the complicated synthesis of this compound. Instead, the 2 recent reports of *sn-2,3-*DAG specificities have relied on using *sn-1,2-2,3-rac-*DAG and compared it with the activity of *sn-1,2-*DAG (Demski et al. 2021; Parchuri et al. 2024). The validity of such assays depends on the fact that the enzyme activity is directly proportional to the amount of DAG substrate, that the TAG lipase gives a 1:1 ratio of *sn-1,2-*DAG and *sn-2,3-*DAG, and there is no interference between enantiomers governing the activity. In this work, we have developed a method to synthesize *sn-2,3-*DAG and used it in our enzyme assays of Arabidopsis DGAT1 and DGAT2. The astonishing results show that DGAT1 and DGAT2 have strict and totally opposite enantiomeric preferences, indicating that DGAT2 has no role in de novo TAG synthesis in Arabidopsis, but might be involved in TAG remodeling. In this context, it is interesting to note that overexpression of AtDGAT2 in an Arabidopsis *dgat1* knockout led to a pronounced increase in 18:1 FA and decrease in 18:3 FA in TAG without a significant change in total oil content (Regmi et al. 2020).

## Materials and methods

### Chemical and reagents

Nonradiolabeled FAs were acquired from Larodan Fine Chemicals (Malmö, Sweden). Glycero-3-phosphocholine (GPC), phospholipase C (from *Clostridium perfringens*), nonradiolabeled TAG, TAG lipase (from *Rhizomucor miehei*), and CoA were obtained from Sigma Aldrich (St. Louis, MO, USA). *sn-1*-6:0-Lyso PC was purchased from Avanti Research (Alabama, USA). Radiolabeled fatty acids were sourced from American Radiolabeled Chemicals (St. Louis, MO, USA) and PerkinElmer (Shelton, USA).

### Synthesis of substrates and standards

Long chain *sn-1,2-*DAGs (*di*-18:1, *di*-18:2, *di*-18:3) were synthesized by acylation of glycero-3-phosphocholine (GPC) with trifluoroacetic acid anhydride of specific FA (Kanda and Wells 1981), which was separated on silica 60 TLC plates (Merck) in chloroform:methanol:acetic acid:water (85:15:10:3.5, v/v/v/v), scraped off and extracted into chloroform with the (Bligh and Dyer 1959) method. PC was treated with phospholipase C in 0.1 M borate buffer, pH 7.4, to produce *sn-1,2-*DAG, which was subsequently separated by silica 60 TLC plates in heptane:diethyl ether:acetic acid, (70:30:1, v/v/v) and extracted into chloroform with the (Bligh and Dyer 1959) method.

*sn-1,2-2,3*-*rac*-18:1 DAG was obtained by treating 15 µmol of *tri-*18:1 TAG with 10 uL of TAG lipase (from *Rhizomucor miehei*) enzyme in 1 mL of sodium borate buffer (containing 10 mM CaCl_2_, pH 7.4) for 10 min and separating DAG on silica 60 TLC plates in heptane:diethyl ether:acetic acid, (60:40:1, v/v/v). DAG was eluted and extracted into chloroform using the (Bligh and Dyer 1959) method. Changing the amount of TAG, lipase enzyme and incubation time had little effect on the ratio between *sn-2,3* and *sn-1,2-*DAG enantiomers with *sn-2,3* enantiomers making up about 85-91% and *sn-1,2-*DAG 9-15%.

[^14^C]-labeled acyl-CoA (18:1, 18:2, 18:3) were synthesized as described by (Sanchez et al. 1973).

TAG standards used for selectivity assay were synthesized by treating 15 µmol of TAG (*tri*-18:1, *tri-*18:2, *tri-*18:3 TAG) with 10 µL of *Rhizomucor miehei* TAG lipase in 1 mL of sodium borate buffer (containing 10 mM CaCl_2_, pH 7.4) for 10 min, which resulted in the degradation of TAG into DAG and MAG. MAG and DAG were separated on a silica 60 TLC plates in heptane:diethyl ether:acetic acid, (60:40:1, v/v/v), scraped off and extracted in chloroform and separated with the (Bligh and Dyer 1959) method. MAG and DAG were subjected to acylation with anhydride of the desired FA (10 µmol) to obtain TAG of the desired FA composition to be used as standards.

*sn-1*-6:0-Lyso PC (LPC) was used as the starting substrate for *sn-2,3-*18:1 DAG synthesis. Then 30 µmol of *sn-1*-6:0-LPC was subjected to acylation with trifluoro-anhydride of 18:1 FA (20 µmol 18:1 FA and 120 µL of trifluoride anhydride for 30 min in room temperature), resulting in the formation of *sn-1-*6:0*-sn-2-*18:1 PC, which was treated further with phospholipase C to obtain *sn-1-*6:0*-sn-2-*18:1 DAG. This DAG was acylated with anhydride 18:1 FA to obtain *sn-1-*6:0*-sn-2,3-*18:1 TAG, which was separated on silica 60 TLC plates in heptane:diethyl ether:acetic acid, (70:30:1, v/v/v) and eluted from the gel. This TAG was then degraded using 10 µL of *Rhizomucor miehei* TAG lipase in 1 mL of sodium borate buffer containing 10 mM CaCl_2_, pH 7.4 for 10 min to produce *sn-2,3-*18:1 DAG along with *sn-1-*6:0*-sn-2-*18:1 DAG. The TAG lipase reaction product was separated on silica 60 TLC plates in heptane:diethyl ether:acetic acid, (70:30:1, v/v/v) by developing it twice in the same mobile phase and the *sn-2,3-*18:1-DAG, which was separated from *sn-1-6:0-sn-2-*18:1-DAG, was eluted from the gel and utilized in the assays. It should be noted that the 6:0-LPC contained about 10% of *sn-2*-LPC and thus the DAG produced also contained 10% of *sn-1-*18:1-*sn-2-*6:0 DAG which later got acylated to *sn-1-*18:1*-sn-2-*6:0-*sn-3-*18:1 TAG. However, these compounds were separated from the *sn-2,3*-18:1-DAG in the last TLC separation. Analyses of the final DAG product by chiral HPLC showed that it contained 92% *sn-2,3-*DAG and 8% *sn-1,2-*DAG. The acylation of the *sn-1,2-*DAG at position *sn-3* with 18:1 also yielded some TAG with *tri-*18:1-TAG and TAG with two 6:0 acyl groups through re-arrangement occurring during the anhydride reaction. The *tri-*18:1 TAG was poorly separated from *sn-1*-6:0-*sn-2,3*-18:1 TAG on the TLC in heptane:diethyl ether:acetic acid, (70:30:1, v/v/v), when large quantities were applied to the plate. We believe that the 8% *sn-1,2-*18:1 DAG contamination primarily originated from some *tri-*18:1 TAG in the *sn-1*-6:0*-sn-2,3-*18:1 TAG band removed from TLC. It might be possible to minimize this contamination by double developing the TLC plate in the same solvent when isolating TAG after the acylation of *sn-1,2-*DAG at the *sn-3* position.

Quantification of all the substrates and standards synthesized in-house was carried out by methylation of FA to fatty acid methyl esters (FAME) with 2% (v/v) sulfuric acid in methanol and quantified using an Agilent Technologies 8860 gas chromatograph (8 CP-wax 58 (FFAP-CB) column) with C17:0 methyl ester as internal standard.

### Yeast cloning, transformation, and microsomal preparations

The Gateway cloning system was used to recombine *S. cerevisiae* codon-optimized Arabidopsis DGAT1 (accession number: NM_127503.3) and DGAT2 (accession number: NM_115011.3) genes obtained from IDT. Initially, the genes were cloned into entry vector (pDONOR221) followed by recombination into the yeast destination vector (pYES2-DEST52), following the guidelines provided by Invitrogen. The quadruple yeast mutant H1246 (dga1Δ lro1Δ are1Δ are2Δ) (Sandager et al. 2002) was transformed with AtDGAT1 and AtDGAT2 constructs in the final destination plasmids with GAL1 promoter.

Recombinant yeast cells were grown in uracil-dropout YNB medium with 2% glucose overnight at 30 ℃. The following day, the yeast was transferred to uracil-dropout YNB medium with 2% galactose and grown for 24 h, leading to the overexpression of the gene and protein synthesis. The microsomal membranes were extracted by homogenizing and ultracentrifugation of the harvested cells under cold conditions, as described by (Lager et al. 2013). The protein concentration of the extracted microsomal membranes was estimated using Pierce BCA Protein Assay kit (Thermo Scientific).

### Enzyme assays

Yeast microsomal membrane (corresponding to 40 µg protein) was diluted in 86 µL of 50 mM HEPES buffer, 5 mM MgCl_2_ (pH 7.2). *Di-*18:1 DAG, *di*-18:2 DAG, *di-*18:3 DAG (20 nmol) dissolved in 4 µL DMSO was added to the microsomal preparation in buffer during vigorous mixing on a vortex for 40 s, followed by the addition of 5 nmol of radiolabeled acyl-CoA (10 µL, 10000 cpm/nmol) (18:1-CoA, 18:2-CoA, 18:3-CoA) diluted in water with 0.1 mg BSA. The reaction mixture was incubated for 8 min (DGAT1) and 30 min (DGAT2) at 30℃, 1200 rpm. The reaction was terminated by adding 500 µL of methanol:chloroform (1:1, v/v) and 120 µL of 0.15 M acetic acid, and lipids were extracted into chloroform. Then one-fifth of the chloroform phase containing the extracted lipid (50 µL) was taken and measured for radioactivity in a Hidex 300 SL Liquid Scintillation Counter (LSC). The remaining fraction was separated on a silica 60 TLC plates in heptane:diethylether:acetic acid, (70:30:1, v/v/v), visualized with iodine, and TAG spots were scraped off and measured by LSC. The amount of TAG produced was calculated using the percentage of radioactive TAG and total radioactivity measured in the chloroform phase in the LSC. The TAG produced by the endogenous DAG in microsomal preparation was considered as the background by running blank assay with DMSO (4 uL) without DAG and this value was subtracted from the assay values with added DAG. The background ranged from 70 to 200 pmol for DGAT1 and 35 to 190 pmol for DGAT2 depending on the acyl donor used, with 18:1-CoA producing the least and 18:3-CoA producing the most endogenous TAG.

The same methodology was used for stereospecificity assay with substrates including *sn-1,2-*18:1 DAG, *sn-2,3-*18:1 DAG and racemic mixture of *di*-18:1 DAG, with [^14^C]18:3-CoA as acyl donor. An inhibition study was conducted using the same approach, in which the concentration of *sn-2,3*-18:1 DAG varied (0, 4, 8, 12, 16, and 20 nmol) while *sn-1,2*-18:1 DAG remained at constant concentration of 20 nmol for DGAT1 assays, whereas for DGAT2 inhibition assay, concentration of *sn-1,2*-18:1 DAG varied (4, 8, 12, 16, and 20 nmol) while *sn-2,3*-18:1 DAG remained constant (20 nmol). No 0 nmol *sn-1,2-*18:1 DAG control was performed in the DGAT2 inhibition assay due to the unavailability of pure *sn-2,3*-DAG.

Enzyme selectivity assay was conducted as described in the single substrate assay except that 2 acyl acceptors (DAGs) and 2 donors (acyl-CoA) were used in equal amounts, corresponding to a total of 20 and 5 nmol, respectively. One-fifth of the chloroform phase containing the extracted lipid (50 µL) was taken and measured for radioactive amount in LSC. The remaining fraction was separated on reverse-phase thin-layer chromatography (RP-TLC) plates (Silica Gel 60 RP-18 F_254_s) in acetonitrile:tetrahydrofuran (60:40, v/v) solvent system. TAG standards were co-spotted along with the samples to identify and distinguish the various TAG molecular species formed. The TAGs were visualized by placing the TLC plate in iodine vapor. The TAG spots were scraped off and quantified using LSC as described above.

### High pressure liquid chromatography

Samples were analyzed with a Shimadzu LC-2030C 3D (Shimadzu Corporation, Kyoto, Japan) using a chiral stationary normal phase column (CHIRALCEL OD-H, silica gel coated with cellulose-tris(3,5-dimethylphenylcarbamate)), 5 µm particle size, 4.6mm × 250 mm, Daicel Chemical, Tokyo, Japan). The mobile phase was n-hexane:2-propanol (99:1) at flow rate of 1.0 mL/min at 25 °C. A 5 µL aliquot of the sample (containing 10–30 µg of *sn-1,2-* and/or *sn-2,3-*DAGs per 5 µL n-hexane) was injected and ran for 45 min. The peaks were detected using the integrated PDA detector (LC2030 3D system, Shimadzu Corporation, Kyoto, Japan) and detected at 202 nm. The peaks were integrated and the enantiopurity was calculated by taking the peak area divided by the total peak area of all DAG peaks.

### Statistical analysis

All samples were run in biological triplicates and are reported as average ± standard deviation (SD). Statistical analyses and graphical representations were done using GraphPad Prism, version 10.0.0 for Windows, including *t*-tests, as well as 1-way ANOVA followed by post-hoc Tukey's test or Dunnett's multiple comparison test.

## Results

### Substrate specificity of Arabidopsis DGAT1 and DGAT2

For the biochemical characterization of the enzymes, we overexpressed codon-optimized AtDGAT1 and AtDGAT2 genes in a yeast mutant strain H1246 that is inherently deficient in the ability to produce TAGs. The substrate specificity of AtDGAT1 and AtDGAT2 was determined using microsomal preparations of recombinant yeast with both mono and polyunsaturated acyl acceptors and [^14^C]acyl-CoA donors. Blank assays were performed by adding DMSO instead of DAG to account for the background, which are TAGs formed with the endogenous DAG present in microsomal preparation. Linear kinetics (Fig. S1) was performed to optimize the assay time, and the results showed that 8 min for AtDGAT1 was within the linear activity of the enzyme; the results are presented as pmol of TAG formed per mg of microsomal protein per minute. Interestingly, AtDGAT2 exhibited no detectable activity until after 30 min with sn-*1,2-*DAG substrate (Fig. S2); hence, DGAT2 assays were conducted for 30 min, and the results are presented as pmol of TAG formed in the assay.

**Figure 1:**
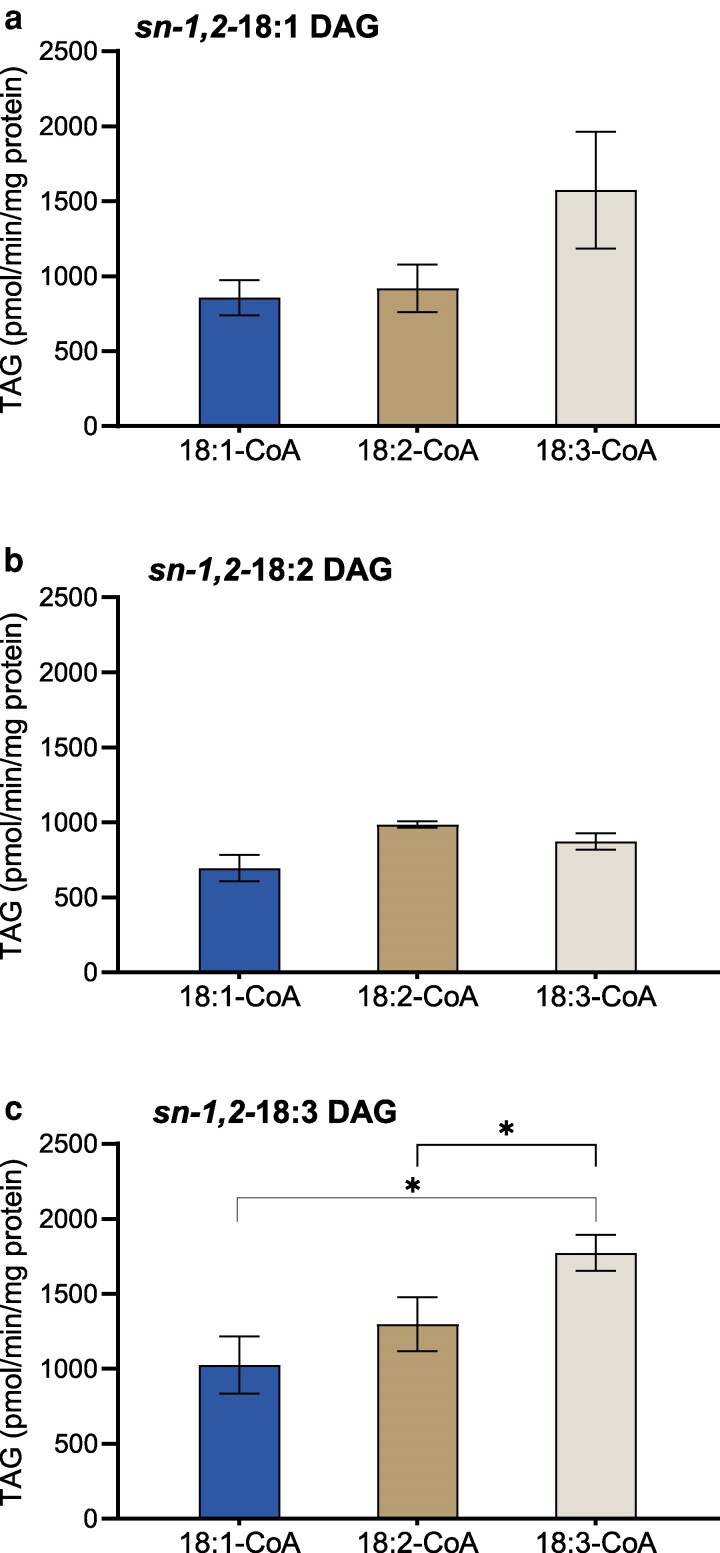
Arabidopsis DGAT1 specificity assay in vitro. Acyl-CoA specificity of the enzyme in microsomal preparations of yeast expressing DGAT1 when incubated with different acyl acceptors (20 nmol): a) *sn-1,2-*18:1 DAG, (b) *sn-1,2-*18:2 DAG, and (c) *sn-1,2-*18:3 DAG. The acyl donors (5 nmol) used are ^14^C-labeled 18:1-, 18:2- and 18:3-CoA, denoted in the *x*-axis. Average value shown ± SD, n = 3 replicates. * denotes significant difference at *P* ≤ 0.05 in 1-way ANOVA followed by post-hoc Tukey test.

The DGAT1 specificity assay using acyl donors and acyl acceptors with 18 carbon FA with different degrees of unsaturation (18:1, 18:2, and 18:3) showed that DGAT1 accepted all tested *sn-1,2-*DAG species at similar levels, with no or small statistical difference between different acyl acceptors (Fig. 1). However, differences in acyl donor specificity were observed within each DAG species. DGAT1 activity increased with the increase in the degree of unsaturation of acyl donors when supplied with *di-*18:1 and *di-*18:3 DAGs (Fig. 1a and 1c). The highest enzyme activity was seen with *di-*18:3 DAG and [^14^C]18:3-CoA, resulting in the formation of trilinolenate (Fig. 1c). When *di-*18:2 DAG was used as an acyl acceptor, AtDGAT1 did not significantly discriminate between the 3 tested acyl donors (Fig. 1b).

**Figure 2:**
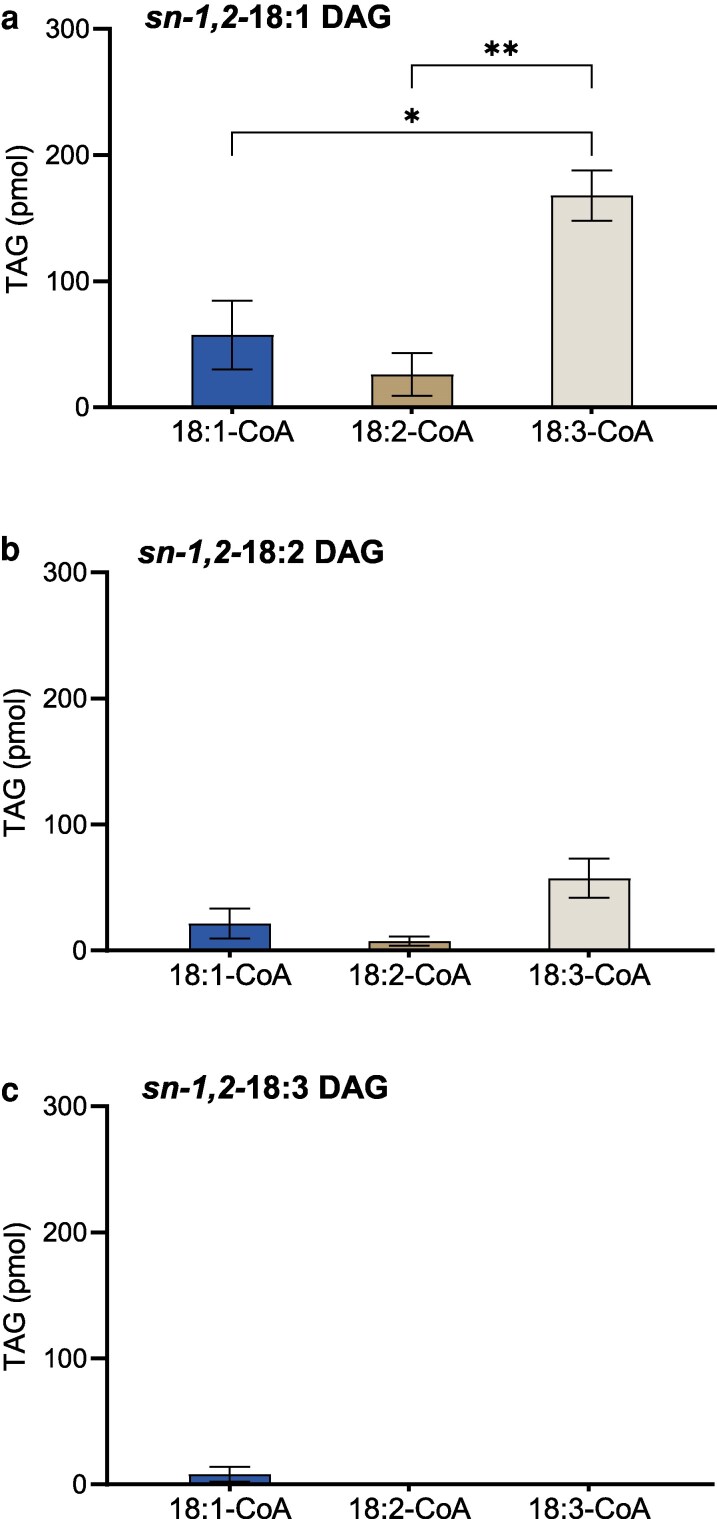
Arabidopsis DGAT2 specificity assay in vitro. Acyl-CoA specificity of the enzyme in microsomal preparations of yeast expressing DGAT2 when incubated with different acyl acceptors (20 nmol): a) *sn-1,2-*18:1 DAG, b) *sn-1,2-*18:2 DAG, and c) *sn-1,2-*18:3 DAG. The acyl donors (5 nmol) used are ^14^C-labeled 18:1-, 18:2-, and 18:3-CoA, denoted in the *x*-axis. Average value shown ± SD, n = 3 replicates. * and ** denote significant difference at *P* < 0.05 and *P* < 0.01, respectively, in 1-way ANOVA followed by post-hoc Tukey test.

AtDGAT2 showed no or minimal activity over background except for *di-*18:1 DAG and *di-*18:2 DAG with [^14^C]18:3-CoA as acyl donor (Fig. 2).

### Selective substrate utilization by Arabidopsis DGAT1

In vitro enzyme assays are effective for studying enzyme specificity but provide limited insights into the enzyme's substrate utilization characteristics, as they do not replicate the complex conditions present in the ER where the DGATs may be exposed to a mixture of substrates. The availability of DAG and acyl-CoA species in the ER and their FA composition play a key regulatory role in TAG biosynthesis by DGATs. Therefore, to understand the selectivity properties of DGAT1, an in vitro competition assay was carried out using yeast microsomal preparations.

DGAT1 was incubated with 2 acyl acceptors and 2 acyl donor combinations, forming 4 possible TAG molecular species. Three assays were carried out with varying combinations of non-radioactive acyl acceptor (DAG) and radioactive donor ([^14^C]acyl-CoA) molecules: (A) *di-*18:1 and *di-*18:2 DAG with 18:1- and 18:2-CoA; (B) *di-*18:2 and *di-*18:3 DAG with 18:2- and 18:3-CoA; and (C) *di-*18:1 and *di-*18:3 DAG with 18:1- and 18:3-CoA. All possible TAG species formed from the combinations of acyl acceptors and acyl donors were separated by reverse phase thin layer chromatography (RP-TLC) based on the number of double bonds present in the FA and were identified with the help of TAG standards (A picture of the TLC plate with the migration patterns of TAG standards is provided in Fig. S3). The radioactivity of each of the TAG species was measured using liquid scintillation counter (LSC). Blank assays were carried out with no addition of DAG to measure the background and were subtracted from the value obtained from corresponding substrate assays. Competition assay was not conducted with AtDGAT2 due to its inherently low activity with single substrates.

**Figure 3:**
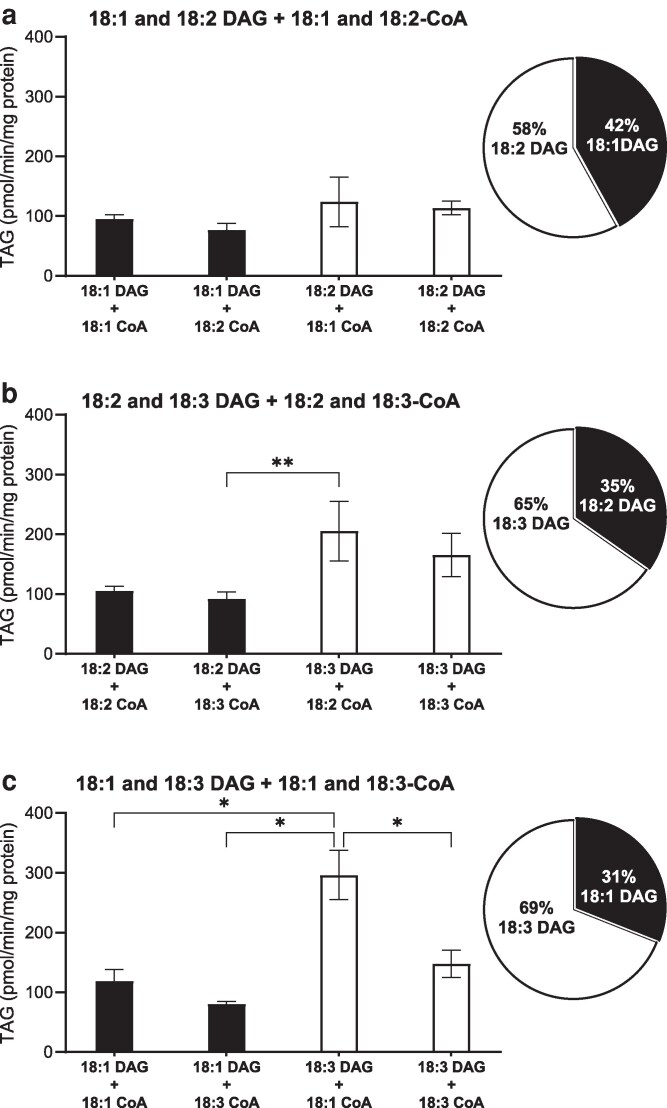
[^14^C]acyl-CoA and DAG selectivity of AtDGAT1. The microsomal preparation of the yeast expressing Arabidopsis DGAT1 was incubated with a mixture of equal amounts of acyl acceptors (totaling to 20 nmol) and ^14^C-labeled donors (totaling to 5 nmol): a) *di*-18:1 and *di-*18:2 DAG with 18:1- and 18:2-CoA. b) *di*-18:2 and *di*-18:3 DAG with 18:2- and 18:3-CoA. c) *di*-18:1 and *di*-18:3 DAG with 18:1- and 18:3-CoA. Each bar is denoted with the DAG and acyl-CoA combination resulting in the formation of that TAG molecular species. The corresponding pie charts summarizes the DAG selectivity pattern of DGAT1, illustrating the acyl acceptors preference of DGAT1. Average value shown ± SD, n = 3 replicates. * and ** denote significant difference at *P* < 0.05 and *P* < 0.01, respectively, in 1-way ANOVA followed by post-hoc Tukey test.

Figure 3a clearly shows that DGAT1 did not demonstrate significant preference between 18:1 and 18:2 DAGs and 18:1- and 18:2-CoA derivatives, resulting in relatively equal proportions of all 4 TAG species formed. Of the DAG utilized, 58% was derived from 18:2 DAG, while the remaining 42% was from 18:1 DAG. When supplied with 18:2 and 18:3 FA containing acyl acceptors and donors (Fig. 3b), DGAT1 exhibited a preference for 18:3 DAG (65%) over 18:2 (35%), with most TAGs formed being 18:3,18:3,18:2 TAG (36%) followed by *tri-*18:3 TAG (29%). When looking into the acyl donor selectivity, a slight preference for 18:2 (55%) over 18:3 (45%) acyl-CoA was observed, but this difference was not statistically significant. In Figure 3c, we observed that when DGAT1 was supplied with equimolar amounts of 18:1 and 18:3 DAGs and acyl-CoAs, the enzyme preferentially utilized 18:3 DAG (69%) over 18:1 DAG (31%). Additionally, there was a statistically significant preference for the monounsaturated acyl donor (18:1-CoA) (64%) over 18:3-CoA (36%). When supplemented with a mixture of substrates and acyl donors, DGAT1 clearly exhibited a higher preference for PUFA containing DAG as the acyl acceptor. Conversely, in the case of acyl donors, DGAT1 preferred less unsaturated acyl-FAs over more unsaturated ones for esterification at *sn-3*. Interestingly, in the specificity study, DGAT1 showed 1.7 times higher activity with *di-*18:3 DAG when paired with 18:3-CoA compared with 18:1-CoA (Fig. 1c). However, in the selectivity study, DGAT1 preferred 18:1-CoA over 18:3-CoA (Fig. 3c), highlighting its selectivity nature over its specificity for the substrate. However, with *di-*18:2 DAG, DGAT1 equally utilized all 3 acyl donors even in the selectivity study, which is consistent with the results of the specificity data (compare Figs. 1 and 3).

**Figure 4:**
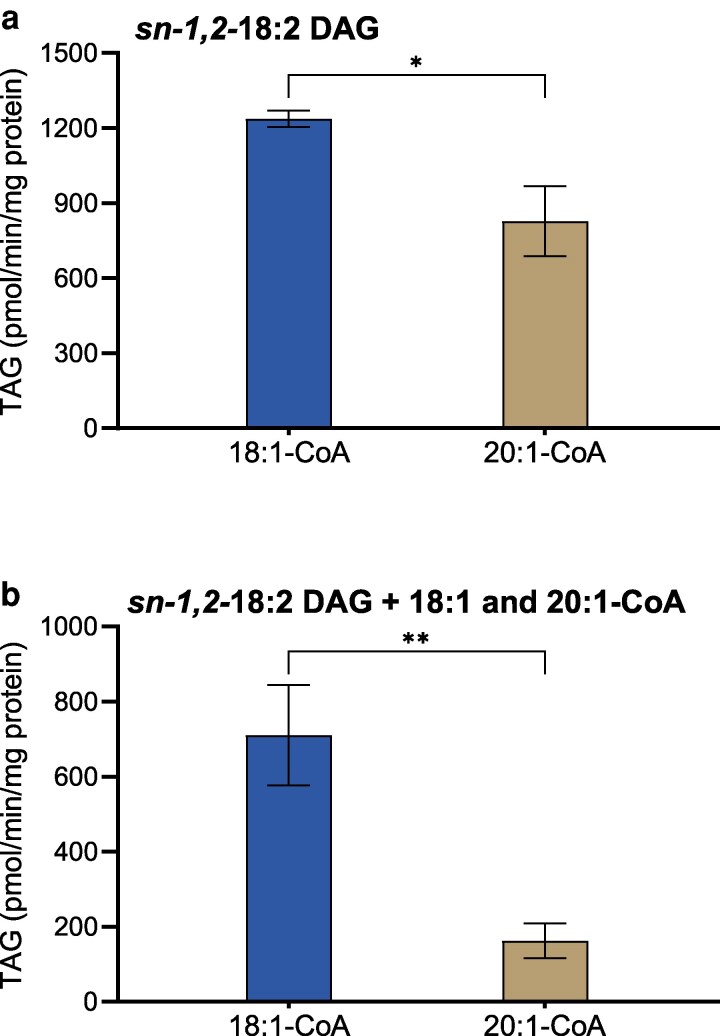
Acyl-CoA specificity and selectivity of AtDGAT1 toward 18:1 and 20:1-CoA. Microsomal fractions from yeast expressing AtDGAT1 were incubated with *di-*18:2 DAG and ^14^C-labeled 18:1 and 20:1 acyl-CoA. a) Acyl-CoA specificity was determined using 18:1 or 20:1-CoA individually. b) Acyl-CoA selectivity was determined using an equimolar mixture of 18:1 and 20:1 acyl-CoA (total 5 nmol). Average value shown ± SD, n = 3 replicates. * and ** denote significant difference at *P* < 0.05 and *P* < 0.01, respectively, determined by unpaired *t* tests (a) and paired *t* tests (B).

Because PC-derived DAG, which is preferentially used by DGAT1, contains low amounts of 20:1 FA, DAG species with 20:1 were not included in the primary experiments. Since 20:1 FA is a dominating acyl group in the *sn-3* position of Arabidopsis TAG, it indicates that 20:1-CoA is preferentially acylated over other acyl groups. To access this, a separate experiment was conducted using *di-*18:2 DAG and 18:1-CoA or 20:1-CoA or a 1:1 mixture of these acyl-CoA (Fig. 4). DGAT1 incorporation of 18:1-CoA was ∼1.5-fold faster than 20:1-CoA when supplied individually (Fig. 4a). In the acyl-CoA selectivity study (Fig. 4b), the preference for 18:1-CoA was even more pronounced, with ∼4-fold more 18:1 incorporated than 20:1 at the *sn-3* position of *di-*18:2 DAG.

### Enantiomeric specificity of Arabidopsis DGAT1 and DGAT2

Recent studies in plant lipid research have provided insights into a “TAG remodeling” pathway that may facilitate the availability of a pool of enantiomeric distinct DAG species available for TAG biosynthesis. To further understand and expand our knowledge on this emerging concept, we chemically synthesized *sn-2,3-*18:1 DAG, which is hypothesized to be the by-product of TAG degradation by TAG lipase enzymes, to investigate if Arabidopsis DGATs accept this enantiomer of DAG to synthesize TAG. The experiment was planned in such a manner that assays were carried out with 20 nmol of *sn-1,2-*18:1 DAG, 10 nmol of *sn-1,2-*18:1 DAG, 20 nmol of a racemic mixture of *di-*18:1 DAG (supposedly containing both the enantiomers obtained from TAG lipase treated *tri-*18:1-TAG), and 20 nmol of *sn-2,3-*18:1 DAG. [^14^C]18:3-CoA was used as the acyl donor due to its highest activity with *sn-1,2-*18:1 DAG in both DGAT1 and DGAT2 (Figs. 1a and 2a).

**Figure 5:**
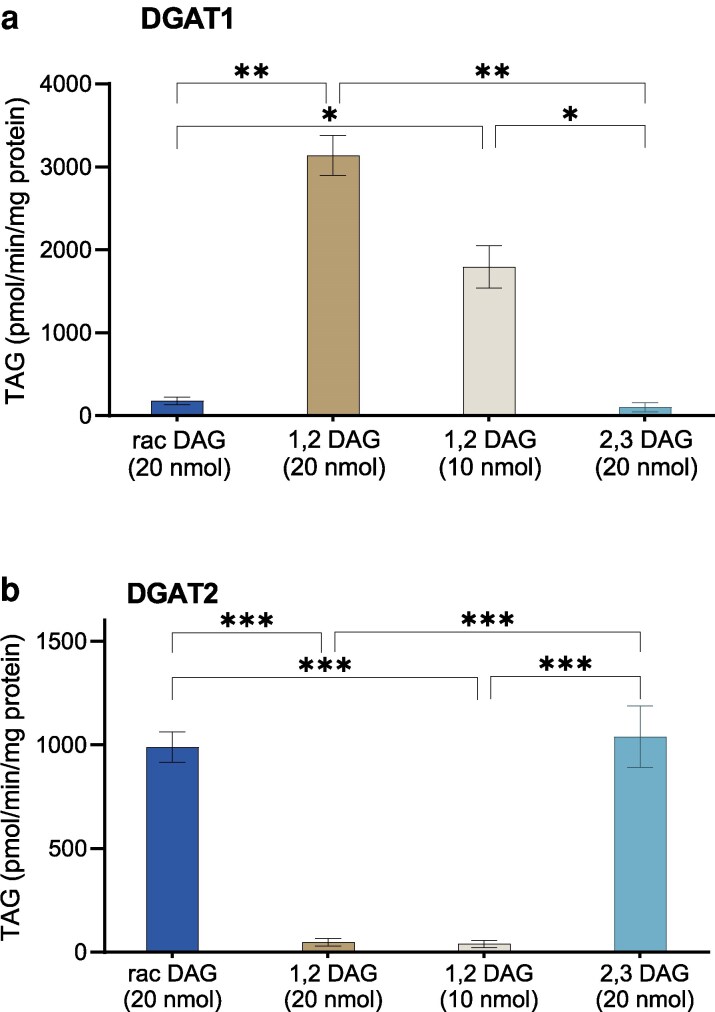
Enantiomeric specificity of DGAT1 and DGAT2 in vitro. Microsomal preparations from yeast expressing Arabidopsis DGAT1 or DGAT2 were incubated with different enantiomers of *di-*18:1 DAGs and [^14^C]18:3-CoA, as indicated in Figure for (a) DGAT1 and (b) DGAT2. The acyl acceptors used were rac DAG, *1,2-2,3-rac-*18:1 DAG, which consist of *sn-2,3-*DAG (91%) and *sn-1,2-*DAG (9%); 1,2 DAG, *sn-1,2-*18:1 DAG (100% pure); 2,3 DAG, *sn-2,3-*18:1 DAG, which consist of *sn-2,3*-DAG (92%) and *sn-1,2*-DAG (8%). The average values are presented ± SD, n = 3 replicates. *, **, and *** denote significant difference at *P* < 0.05, *P* < 0.01, and *P* < 0.001, respectively, in 1-way ANOVA followed by post-hoc Tukey test.

To our surprise, DGAT1 showed hardly any activity with either *rac-*18:1 DAG or *sn-2,3-*18:1 DAG, whereas TAG was produced with both concentrations of *sn-1,2-*18:1 DAG (Fig. 5a). Theoretically, if DGAT1 is only specific to *sn-1,2-*DAG of the enantiomers of DAG, then the amount of TAG produced with 10 nmol of *sn-1,2* DAG and 20 nmol of *sn-1,2-2,3-rac* DAG would be the same. DGAT2 was highly specific to *sn-2,3-*18:1 DAG with 18:3-CoA as an acyl donor. Similar activity was also observed for DGAT2 with racemic 18:1-DAG produced from *tri-*18:1-TAG (Fig. 5b).

**Figure 6:**
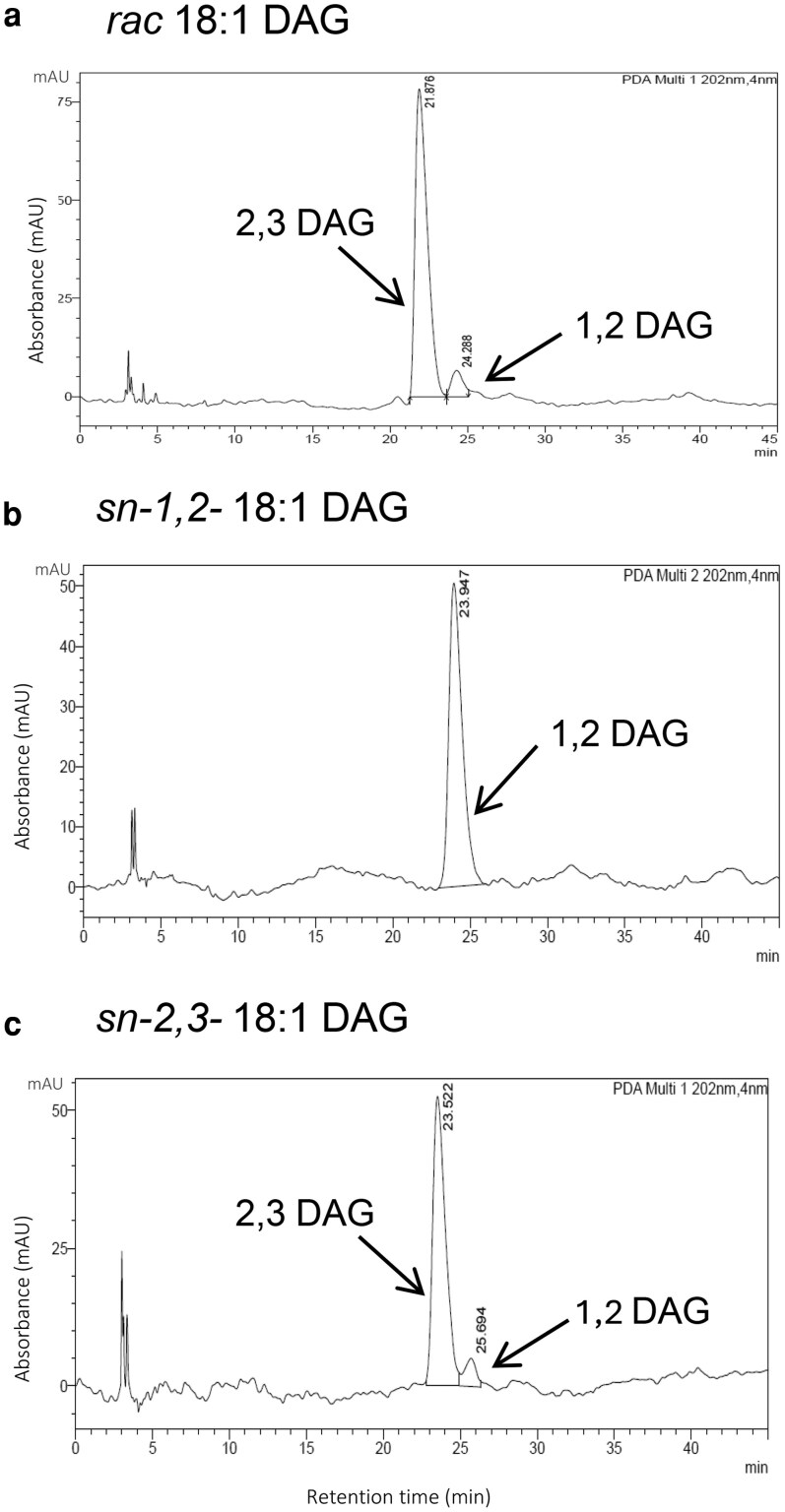
Chiral phase HPLC chromatogram of acyl acceptors (DAG). Chromatograms of (a) *rac*-18:1 DAG, (b) *sn-1,2-*18:1 DAG, and (c) *sn-2,3-*18:1 DAG separated on a chiral phase column to separate the enantiomers *sn-1,2* and *sn-2,3* DAG as distinct peaks. *Rac-*DAG contained 91% sn-1,2 and 9% *sn-2,3* DAG. *sn-1,2-*18:1 DAG was 100% pure. In-house synthesized *sn-2,3-*18:1 DAG had 92% *sn-2,3* and 8% *sn-1,2* DAG. Abbreviation: DAG, diacylglycerol.

In view of the outcome of these experiments, we used high pressure liquid chromatography (HPLC) to separate the 2 enantiomers by chiral chromatography. We could show that the *sn-1,2-*DAG used was 100% pure, whereas our synthesized *sn-2,3* DAG contained 8% of *sn-1,2*; to our surprise, the racemic DAG contained 91% *sn-2,3-*enantiomer and only 9% of *sn-1,2-*enantiomer (Fig. 6). The samples were analyzed in separate runs, which may have contributed to minor shifts in retention time. However, the observed differences fall within the normal margin of error for HPLC methodology, and the order of elution of the DAG enantiomers remained consistent across all analyses.

**Figure 7:**
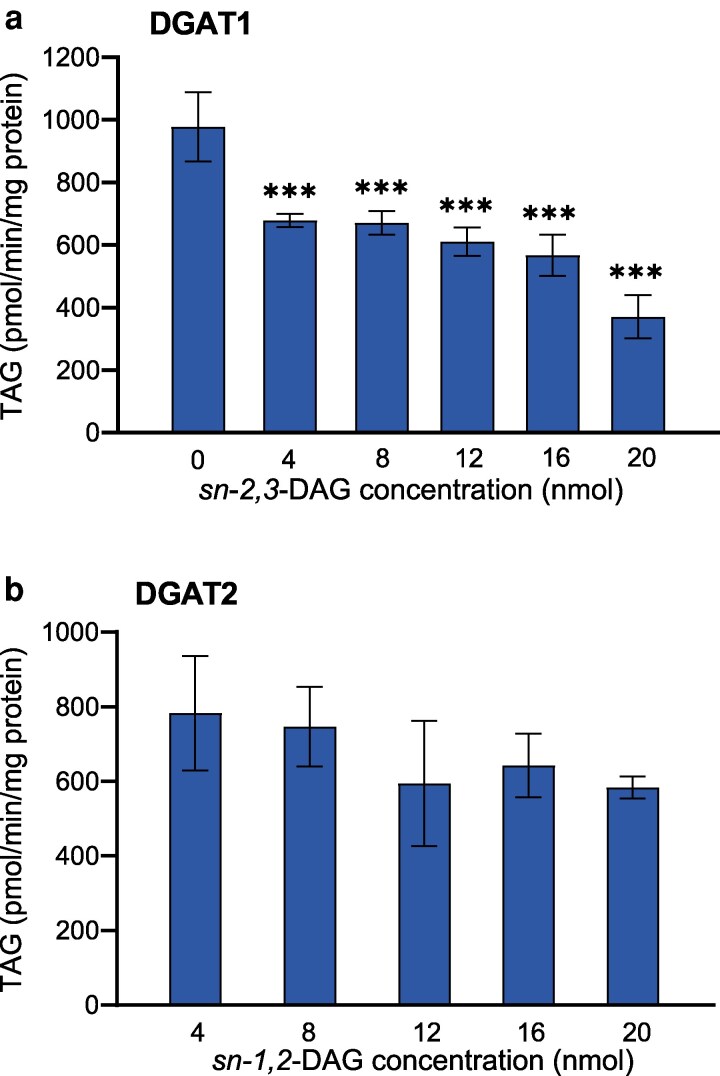
Mixed enantiomer effects on AtDGAT1 and AtDGAT2 activity. Microsomal preparations of yeast expressing (a) DGAT1 and (b) DGAT2 were incubated with different concentrations of *sn-2,3-*18:1 DAG (a) and *sn-1,2-*18:1 DAG (b) with the amounts of the varying DAG denoted along the X-axis. The other enantiomer DAG was kept constant at 20 nmol. [^14^C]18:3-CoA was used as acyl donor. The acyl acceptors used were *sn-1,2-2,3-rac-*18:1 DAG constituted of *sn-2,3*-DAG (85%) and *sn-1,2*-DAG (15%); *sn-1,2*-18:1 DAG (100% pure). The average values are presented ± SD, n = 3 replicates. *** indicates significant differences (*P* < 0.001) from the first column based on 1-way ANOVA followed by Dunnett multiple comparison test.

To explore the extent of inhibition shown by the *sn-2,3-*DAG, we carried out DGAT1 assays with 20 nmol of *sn-1,2-*18:1-DAG mixed with different concentrations of *sn-2,3-*18:1 DAG (0, 4, 8, 12, 16, and 20 nmol). This study (Fig. 7a) showed 50% reduction in TAG formation when 20 nmol of *sn-2,3*-18:1 DAG was added along with 20 nmol of *sn-1,2-*18:1 DAG, when compared with the amount of TAG formed with 20 nmol of *sn-1,2*-18:1 DAG alone as the acyl acceptor. It should however be noted that the *sn-2,3-*DAG also contained 15% *sn-1,2-*DAG, the preferred substrate for DGAT1. When DGAT1 was provided with only *sn-2,3-*DAG preparation containing 8-9% *sn-1,2-*DAG in it, no significant activity was observed (Fig. 5a). For DGAT2, no significant reduction in TAG formation was detected when adding sn-*1,2*-18:1 DAG to 20 nmol of sn-*2,3*-18:1 DAG, although substantial variation was evident across different concentrations of *sn-1,2-*DAG (Fig. 7b).

## Discussion

### Substrate specificity and selectivity of Arabidopsis DGATs have important implications for channeling of DAG species into TAG

A part of this work focuses on the biochemical characterization of Arabidopsis DGAT1 and DGAT2 overexpressed in microsomal preparations from a yeast strain (H1246) devoid of TAG to evaluate how the specificity and selectivity of these enzymes affect the FA composition of the TAG. There are 2 main theories of how DAG acylation enzymes might influence the acyl composition of the synthesized TAG: by enzyme selectivity or by utilization of different DAG pools. The existence of different DAG pools and their utilization in TAG synthesis was suggested based on [^14^C]glycerol and [^14^C]acetate in vivo labeling experiments in soybean (*Glycine max*) and Arabidopsis developing seed embryos (Bates et al. 2009; Bates and Browse 2011; Regmi et al. 2020; Bates and Shockey 2025). In these works, DAG derived from PC by DAG:PC equilibration catalyzed by PDCT was preferentially acylated to TAG over de novo synthesized DAG. However, the apparent spatial separation of DAG pools cannot be easily distinguished from DAG selectivity exhibited by DGATs in the acylation to TAG. Strong DAG specificities and selectivity have been demonstrated by DGATs from Camelina (*Camelina sativa*) and castor bean (Lager et al. 2020; Demski et al. 2022).

Arabidopsis knock-outs of PDAT1 and DGAT2 do not affect the FA composition and content of seed TAG (Mhaske et al. 2005; Zhang et al. 2009), whereas *dgat1* knockdown or knockout led to a 20% to 40% decrease in oil and change in FA composition of TAG (Zou et al. 1999; Zhang et al. 2009; Xu et al. 2012; Regmi et al. 2020). When *dgat1* mutant was combined with RNAi toward PDAT1, TAG content was reduced to 80% compared with the wild type (Zhang et al. 2009). It can be concluded from these studies that DGAT1 is the major, and perhaps only, enzyme contributing to TAG synthesis in Arabidopsis wild type, but in its absence, PDAT1 can partially compensate for TAG biosynthesis. The role of DGAT2 in Arabidopsis remains unclear, although the expression of the gene is relatively high throughout the plant, including in developing seeds (Li et al. 2010). Overexpression of DGAT2 in *dgat1* mutant increased the proportion of 18:1 FA and decreased 18:3 FA in TAG (Jako et al. 2001; Regmi et al. 2020).

In our specificity study, Arabidopsis DGAT1 and DGAT2 activity were evaluated for their preference with different acyl acceptors and donors individually. Notably, DGAT1 demonstrated no preferential discrimination among the acyl acceptors (Fig. 1), indicating its broad-spectrum specificity. The very low activity of Arabidopsis DGAT2 is in line with the low amount of TAG observed when it is expressed in yeast compared with Arabidopsis DGAT1 (Aymé et al. 2014). However, this contrasts sharply with the higher ability of DGAT2, when compared with DGAT1, to produce TAG in *Nicotiana benthamiana* leaves when expressed transiently, as well as in in vitro assays of microsomal preparations of these leaves using *sn-1,2-2,3-rac-*6:0 DAG as acyl acceptor (Zhou et al. 2013). Since the activity of DGAT2 microsomes was low when measured with *sn-1,2-*DAG as acyl acceptor, it was not possible to proceed with selectivity studies with this DGAT isozyme.

The selectivity studies where 2 different acyl acceptors and 2 different acyl-CoA species were mixed (Fig. 3) demonstrated that Arabidopsis DGAT1 preferentially utilized polyunsaturated acyl acceptors over monounsaturated, whereas monounsaturated donors were selected over polyunsaturated donors. Bates and co-authors (Bates et al. 2009; Bates and Browse 2011) showed that most of the TAG is synthesized from PC-derived DAG, which means the PC-derived DAG is likely to have a higher amount of 18:2 and 18:3 than bulk DAG, since PC is the site of PUFA synthesis. Metabolic labeling experiments in developing seeds of Arabidopsis show that 14 out of 15 parts of de novo synthesized DAG are promptly converted to PC-derived DAG (Bates and Browse 2011), meaning that the flux of de novo DAG to PC is much higher than its direct flux to TAG. Our results clearly showed that DGAT1 selectivity toward PUFA containing DAGs could also play a significant role in the rapid utilization of PC-derived DAG in TAG synthesis compared with the bulk DAG pool. However, there is also strong evidence that PC-derived DAG is spatially separated from the bulk DAG pool, forming a TAG yielding metabolon with DGAT1 (Regmi et al. 2020). It should be noted that both the selectivity criteria of DGAT1 and spatial separation of DAG might coexist and affect the composition of TAG in Arabidopsis.

The acyl-CoA selectivity study with 18:1 and 20:1-CoAs (Fig. 4b) showed that DGAT1 preferred 18:1-CoA over 20:1-CoA to acylate the *sn-3* position despite 20:1 FA being the dominating acyl group in the *sn-3* position of Arabidopsis TAG (Taylor et al. 1995). These results indicate that the high 20:1 content at the *sn-3* position of TAG cannot be explained by DGAT1 substrate selectivity. Rather, it likely reflects the abundance of 20:1 within the acyl-CoA pool, which has been reported to predominate among TAG-associated acyl-CoAs during Arabidopsis seed development (Yurchenko et al. 2014). The higher activity of DGAT1 with 18:1-CoA than with 20:1-CoA also explains the lack of reduced TAG synthesis in the *fae1* mutant, in which 18:1 elongation to 20:1 is eliminated (Kunst et al. 1992; James et al. 1995). Although acyl-CoA selectivity was not a primary focus of this study, these findings underscore that the substrate availability, in addition to the intrinsic enzyme specificity, can play a decisive role in governing the TAG composition.

### The enantiomeric specificity of Arabidopsis DGAT1 and DGAT2 reveals that they are nonredundant enzymes using different DAG pools

DAG can come in 2 forms that are mirror images of each other. The DAG synthesized by the G3P pathway (de novo) is always at *sn-1,2* configuration and is utilized in both TAG and membrane lipid synthesis. On the other hand, the mirror image, the enantiomer *sn-2,3*-DAG, can be formed in the cell by removal of the *sn-1* acyl group of TAG (Parchuri et al. 2024) and in vertebrates by acylation of the *sn-2*-monoacylglycerol (MAG) at the *sn-3* position by the monoacylglycerol acyltransferases (MGATs) (Turchetto-Zolet et al. 2011). Information about enantiomeric specificities of DGATs is scarce, and all assays of DGATs have been performed with either *sn-1,2*-DAG or a racemic *sn-1,2-2,3-rac-*DAG. Recently (Demski et al. 2021) and (Parchuri et al. 2024) tried to assess enantiomeric specificities of DGATs with castor DGAT2 and *Physaria fendleri* DGATs by comparing the activities with *sn-1,2*-DAG with the same amounts of *sn-1,2-2,3-rac-*DAG with the assumption that the *sn-1,2-2,3-rac* DAG had equal amounts of *sn-1,2*-DAG and *sn-2,3*-DAG. We prepared *sn-1,2-2,3-rac-*18:1 DAG with *Rhizomucor miehei* lipase acting upon *tri-*18:1-TAG but also prepared *sn-2,3*-18:1 DAG by a newly developed method as described in the Methods section and used it together with *sn-1,2*-18:1 DAG as acyl acceptors to assay Arabidopsis DGAT1 and DGAT2 activities. It should be noted that at that time of the enzyme assays we did not have access to the HPLC chiral phase to determine the relative proportions of the enantiomers in the different DAG preparations. When later analyzing the DAGs with HPLC, we found that the *sn-1,2*-DAG was 100% pure, but the *sn-2,3*-DAG synthesized by us contained about 8% of *sn-1,2*-DAG, and, to our surprise, the *sn-1,2-2,3-rac-*DAG contained 91% *sn-2,3*-DAG and 9% *sn-1,2*-DAG. The *Rhizomucor miehei* lipase has been reported to have a preference for *sn-1* position (Lehner et al. 1993), but such preference was also dependent on the supplier of the lipase (Sonnet 1988). Such high discrepancies between *sn-1,2*-DAG and *sn-2,3*-DAG proportions produced by *Rhizomucor miehei* as we had have not been reported before.

There was a dramatic and opposite effect on Arabidopsis DGAT1 and DGAT2 when using on the one hand *sn-1,2*-DAG and on the other hand *sn-2,3*-DAG and “racemic” DAG. There was no significant activity of DGAT1 with the racemic (91% *sn-2,3*-DAG) and the *sn-2,3*-DAG preparation (Fig. 5a), whereas DGAT2 was essentially inactive with *sn-1,2*-DAG but had high and equal activity with “racemic” DAG and *sn-2,3*-DAG preparation (Fig. 5b). We therefore also tested whether *sn-2,3*-DAG and *sn-1,2*-DAG was inhibiting the DGAT1 and DGAT2, respectively, by adding increasing amounts of *sn-2,3*-DAG to 20 nmol of *sn-1,2*-DAG in assays of DGAT1 and increasing amounts of *sn-1,2*-DAG along with 20 nmol of *sn-2,3*-DAG in assays of DGAT2. Adding 20 nmol of *sn-2,3*-DAG reduced the activity of AtDGAT1 by about 50% (Fig. 7a), but the decrease in activity was not linear with *sn-2,3-*DAG concentration. In case of DGAT2, addition of 20 nmol of *sn-1,2*-DAG along with 20 nmol of *sn-2,3*-DAG did not cause a statistically significant decrease, although the variation at different concentrations of *sn-1,2-*DAG were high (Fig. 7b). The cause of the decrease in the activity of DGAT1 by *sn-2,3-*DAG is unclear. It should be noted that the *sn-2,3-*DAG preparation contained 15% of *sn-1,2-*DAG, the effect of which on the rate of activity in the experiment is unknown. Therefore, repeating the experiment with 100% pure *sn-2,3-*DAG, if such purity can be achieved, should clarify this point.

Our results suggest that Arabidopsis DGAT1 is a *sn-1,2*-DAG enantio-specific enzyme, whereas DGAT2 is *sn-2,3*-DAG enantio-specific, or alternatively, that they possess extreme and opposite enantiomeric specificities. We can thereby establish that AtDGAT2 is not involved in de novo TAG synthesis but might be involved, together with a lipase, in the remodeling of TAG at *sn-1* position, similar to what has been suggested by (Parchuri et al. 2024) in the remodeling of TAG by *Pfe*DGAT2 to increase the amount of hydroxy FAs in TAG in *Physaria fendleri*. Thus, remodeling TAG might not be specific to plants that accumulate high amounts of “unusual” FAs in TAG but might be a feature of all oilseed plants. DGAT2 has been shown to utilize the slowly producing DAG pool in Arabidopsis (Regmi et al. 2020). This, combined with our results, where DGAT2 is specific for *sn-2,3*-DAG suggests that the pool of slowly produced DAG might be enriched with *sn-2,3*-DAG enantiomer. These results could be further supported by the late catalytic onset of DGAT2 activity (Fig. S2), which could allow sufficient time for the added *sn-1,2*-DAG to acyl migrate to small amounts of *sn-2,3*-DAG (via *sn-1,3-*DAG) in the aqueous environment during the assay.

The discovery that Arabidopsis DGAT2 is *sn-2,3*-DAG specific explains the contradictory results obtained by expressing the DGAT2 gene in yeast, which yielded only a fraction of TAG in the yeast cells compared with DGAT1 expressed in yeast (Aymé et al. 2014). On the other hand, expressing DGAT2 in leaves of *Nicotiana benthamiana* increased the TAG amounts in the leaves more efficiently than DGAT1 (Zhou et al. 2013), possibly due to more abundance of *sn-2,3*-DAG in these leaves compared with yeast. However, it is apparent that DGAT2 in other plant species can also acylate *sn-1,2*-DAG, in contrast to the Arabidopsis enzyme, as seen with castor DGAT2 (Demski et al. 2022) and Camelina DGAT2 (Lager et al. 2020). It will be interesting to further investigate how DGATs handle enantiomers, an emerging area in TAG biosynthesis, not only in plants, as highlighted in recent publications (Regmi et al. 2020; Parchuri et al. 2024; Bates and Shockey 2025).

DGAT1 and DGAT2 enzymes are both found in nearly all eukaryotes and have evolved independently since the emergence of eukaryotes 2.5 billion years ago (Turchetto-Zolet et al. 2011). The fact that both these enzymes have been maintained through millions of years of evolution strongly suggests that they serve essential, nonredundant functions. While DGAT1 is recognized as the primary contributor to TAG biosynthesis due to its pronounced phenotypic impact on plants, the physiological role of DGAT2 in plants with common fatty acids remains unclear. Therefore, the discovery of the different enantiomeric specificities of Arabidopsis DGAT enzymes may shed light on the respective enzymes' crucial role in all eukaryotes.

## Supplementary Material

kiag234_Supplementary_Data

## Data Availability

All data supporting the findings of this study are included in the article and/or its figures.

## Accession numbers

Sequence data from this article can be found in the GenBank/EMBL data libraries under accession numbers NM_127503.3 (AtDGAT1) and NM_115011.3 (AtDGAT2).
