# Supplementary material for: Opposite diacylglycerol enantiomeric specificities of Arabidopsis DGAT1 and DGAT2 reveal distinct roles in TAG synthesis
Source: Plant Physiol. 2026 Apr 21;201(1):kiag234. doi: 10.1093/plphys/kiag234 (PMC13181396; doi:10.1093/plphys/kiag234)
Supplement: kiag234_Supplementary_Data [file kiag234_supplementary_data.pdf]

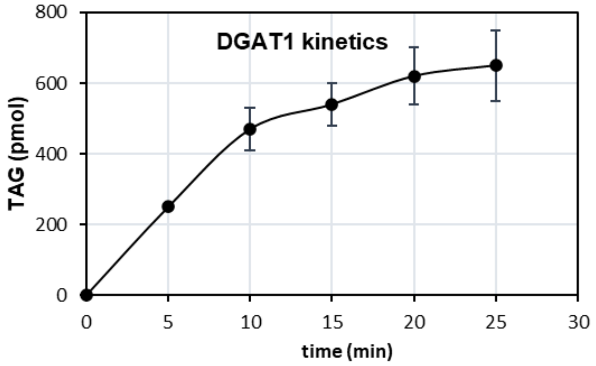

Supplementary Figure S1: Time course of DGAT1 activity. Microsomal preparation (40ug) of yeast expressing Arabidopsis DGAT1 was incubated with *sn-1,2*-18:2 DAG and [^{14}C]18:1-CoA. The average values are presented ± SD, n = 3 replicates.

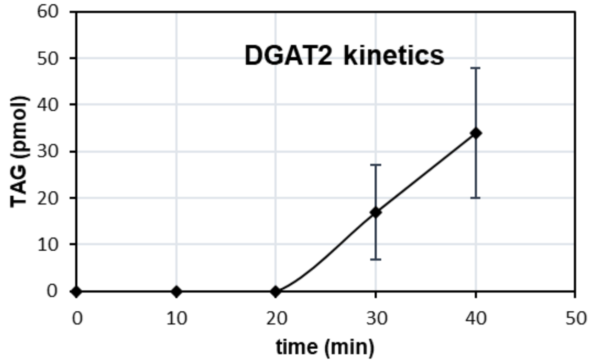

Supplementary Figure S2: Time course of DGAT2 activity. Microsomal preparation (40ug) of yeast expressing Arabidopsis DGAT2 was incubated with *sn-1,2*-18:2 DAG and [^{14}C]18:3-CoA. The average values are presented ± SD, n = 3 replicates.

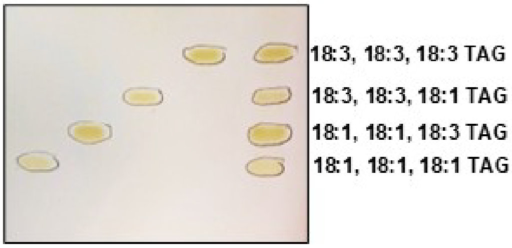

Supplementary Figure S3: TAG standard separation on reverse phase – Thin Layer Chromatography (RP-TLC). Triacylglycerol (TAG) standards, with varying fatty acids (FAs) occupying the glycerol backbone, are separated using reverse-phase thin-layer chromatography (RP-TLC) plates (Silica Gel 60 RP-18 F_{254}s), based on the number of carbon atoms and double bonds in the fatty acid chains. The TLC plate was visualized by placing it in a chamber with iodine vapor. Areas containing the separated TAG species are encircled with pencil and labeled.
